# An Overview of Biosimilars—Development, Quality, Regulatory Issues, and Management in Healthcare

**DOI:** 10.3390/ph17020235

**Published:** 2024-02-11

**Authors:** Filipa Mascarenhas-Melo, Mariana Diaz, Maria Beatriz S. Gonçalves, Pedro Vieira, Victoria Bell, Sofia Viana, Sara Nunes, Ana Cláudia Paiva-Santos, Francisco Veiga

**Affiliations:** 1 LAQV-REQUIMTE, Group of Pharmaceutical Technology, Faculty of Pharmacy, University of Coimbra, 3000-548 Coimbra, Portugal; fveiga@ff.uc.pt; 2 Higher School of Health, Polytechnic Institute of Guarda, 6300-307 Guarda, Portugal; 3 Drug Development and Technology Laboratory, Faculty of Pharmacy, University of Coimbra, 3000-548 Coimbra, Portugal; mariana.guerra.diaz@gmail.com (M.D.); mariabsgoncalves@gmail.com (M.B.S.G.); 4 Institute of Pharmacology & Experimental Therapeutics & Coimbra Institute for Clinical and Biomedical Research (iCBR), Faculty of Medicine, University of Coimbra, 3000-548 Coimbra, Portugal; pedromdvieira96@gmail.com (P.V.); sofia_viana@estesc.ipc.pt (S.V.); or spnunes@fmed.uc.pt (S.N.); 5 CIBB—Center for Innovative Biomedicine and Biotechnology, University of Coimbra, 3004-504 Coimbra, Portugal; 6 Clinical Academic Center of Coimbra (CACC), 3004-504 Coimbra, Portugal; 7 Coimbra Health School, Polytechnic Institute of Coimbra, 3046-854 Coimbra, Portugal; 8 Laboratory of Social Pharmacy and Public Health, Faculty of Pharmacy, University of Coimbra, 3000-548 Coimbra, Portugal; victoriabell@ff.uc.pt

**Keywords:** biosimilars, development approach, interchangeability, quality assessment, regulatory approach

## Abstract

Biological therapies have transformed high-burden treatments. As the patent and exclusivity period for biological medicines draws to a close, there is a possibility for the development and authorization of biosimilars. These products boast comparable levels of safety, quality, and effectiveness to their precursor reference products. Biosimilars, although similar to reference products, are not identical copies and should not be considered generic substitutes for the original. Their development and evaluation involve a rigorous step-by-step process that includes analytical, functional, and nonclinical evaluations and clinical trials. Clinical studies conducted for biosimilars aim to establish similar efficacy, safety, and immunogenicity, rather than demonstrating a clinical benefit, as with the reference product. However, although the current knowledge regarding biosimilars has significantly increased, several controversies and misconceptions still exist regarding their immunogenicity, extrapolation, interchangeability, substitution, and nomenclature. The development of biosimilars stimulates market competition, contributes toward healthcare sustainability, and allows for greater patient access. However, maximizing the benefits of biosimilars requires cooperation between regulators and developers to ensure that patients can benefit quickly from access to these new therapeutic alternatives while maintaining high standards of quality, safety, and efficacy. Recognizing the inherent complexities of comprehending biosimilars fully, it is essential to focus on realistic approaches, such as fostering open communication between healthcare providers and patients, encouraging informed decision-making, and minimizing risks. This review addresses the regulatory and manufacturing requirements for biosimilars and provides clinicians with relevant insights for informed prescribing.

## 1. Introduction

In 1980, biological medicines emerged and began to be developed as a new group of medicines produced in living systems, using biotechnological methods (recombinant DNA technology), which differentiated them from traditional chemical synthesis medicines. These differences led to the need for specific legislation adapted to these new medicines [1,2,3,4].

Several diseases (such as cancer, diabetes, autoimmune diseases, and multiple sclerosis) can be life-threatening. Since they began to be introduced into patient treatment, biological medicines have shown their value as a key health technology, being one of the most promising segments of the pharmaceutical industry, with a vast and growing population benefiting from their use. With the fall of their patents, biosimilars appeared as equivalent alternatives. However, to gain their place in the market, they needed to demonstrate an equal safety profile to their reference biologicals [4,5].

This review article aims to explain the differences between biological medicines and biosimilars and between these and intended copies, biobetters, and standalone biologics. We will provide a detailed explanation of the development and regulatory aspects required for the approval of biosimilars, including an analytical similarity assessment, as well as non-clinical and clinical similarity. In addition, it also focuses on how the safety control of biosimilars is carried out post-marketing (pharmacovigilance) and on the disputes concerning these medicines that still exist (immunogenicity, extrapolation, interchangeability, substitution, and nomenclature). 

This is an innovative publication supported by available reports, composed of an informed and critical discussion that updates and details the main properties of biosimilars and the regulatory and manufacturing processes employed in their development and approval; addresses the post-marketing monitoring of the safety of biosimilars and the main controversies in the use of biosimilars, providing information for clinical decisions regarding the use of biosimilars; and also gives an example of a model for the implementation of biosimilars in a hospital setting. This manuscript stands out as it brings together such relevant and up-to-date information on and discussions of biosimilars.

### Key Points

− The use of biosimilar medicines contributes to greater patient access to biological therapies. However, for this to happen, healthcare professionals and patients must understand the concept of biosimilarity.
− The development of a biosimilar involves conducting a comparability assessment between the intended biosimilar and the reference biological. The primary objective is not to establish their efficacy and safety independently but rather to validate the similarity between the two products.
− Despite their recognized value, biosimilars are still subject to some controversies, such as immunogenicity, interchangeability and substitution, extrapolation, and nomenclature.

## 2. Biological Medicines

The human body continually produces enzymes, hormones, antibodies, and other endogenous substances necessary for its survival. Over time, drug development has tried to compensate, through targeted approaches, for any deficiencies observed in certain diseases or disorders. More than two decades ago, biological medicines were introduced on the market [6]. They are produced from living cells, using biotechnology techniques, unlike common medicines, which are produced from chemical synthesis in the laboratory [7,8,9]. As previously mentioned, biological medicines play a critical role in the treatment of various diseases [6,9].

Biological medicines possess a significantly more intricate and larger structure when compared to small-molecule drugs [4]. Currently, they are divided into three main categories: (1) products with a high correlation to endogenous factors, often used as a substitute therapy; (2) monoclonal antibodies (mAbs) that bind to soluble or cell surface targets, blocking cell signaling pathways and their functional responses; (3) engineered proteins that mimic soluble receptors, receptor antagonists, and fusion proteins. More specifically, biological medicines are produced as hormones (as is the case of insulin, hormone deficiencies, and growth hormones), mAb (e.g., the management of autoimmune diseases and cancer), blood products (e.g., individuals with hemophilia), immunomodulators (e.g., interferon beta, for multiple sclerosis), enzymes (e.g., for the removal of blood clots), and vaccines for the prevention of various diseases [8,10]. 

The production of most biological drugs is conducted through genetically modified host cells, which can be derived from plants, yeasts, bacteria, or animals. Each manufacturer has its own host cell bank, allowing it to produce a distinct cell line. Furthermore, each manufacturer is responsible for its own manufacturing process [10]. First, the genetic code of the chosen protein is identified, which can be a hormone, an antibody, or a blood derivative, among others, and then a DNA sequence is fully synthesized. The resulting genetic code is added to different host cell lines. (e.g., yeast or bacteria) for them to produce this protein. Then, the strain that produces the most effective protein is selected and cultivated in bioreactors, a process called fermentation. Afterward, outside the bioreactor, the separation, purification, and stabilization of the protein occur. Then, it is processed into a drug [11]. 

The problem with biological drugs lies in the fact that their activity can be impaired by the environmental and manufacturing conditions, making it difficult to achieve equivalent purity between different batches [4]. Several factors contribute to this: inadequate selection of the cell line; the biophysical characteristics of proteins; changes in temperature or pH conditions during the cultivation phases; the handling and conservation of the product in the various stages of manufacture; the drug product formulation; the production scale; and the production site. Hence, batch discrepancies are circumvented through the implementation of rigorous manufacturing controls over the cellular systems used [4,8].

Over the past few years, the periods of exclusivity rights to numerous biological medicines have expired. This has led to the introduction and authorization of “biosimilars”, products with strong similarity to other licensed biologicals [4,8,9,12].

### 2.1. Biosimilars

Following the approval of the first biosimilar drug (somatropin) in 2006 by the European Medicine Agency (EMA), the market of biosimilars has seen considerable expansion. Europe approved, for the first time, an mAb biosimilar (infliximab) in 2013. It is known that between 30 and 50% of new medicines approved in Europe are currently biosimilar [13]. By August 2022, 1775 biological products were licensed in the US market, of which 82 were biosimilar [14].

The European Medicine Agency’s guideline states that “a biosimilar is a biological medicinal product that contains a version of the active substance of an already authorised original biological medicinal product (reference medicinal product—RP) in the European Economic Area (EEA)”. It is necessary to establish a comprehensive comparability exercise to determine the similarity to the reference medicinal product in terms of quality characteristics, biological activity, safety, and efficacy [15]. The biosimilar’s pharmacokinetic (PK) and pharmacodynamic (PD) properties are similar to those of an existing biological medicine (i.e., a medicine that has already received approval from the EMA and is used in the EU, according to approved therapeutic indications). When the approved biological medicine is no longer protected by its exclusivity period (generally 10 years) or by a patent, a biosimilar medicine can be introduced on the market [9,16].

Biological medicines, including biosimilars, are known for their complex characteristics and high heterogeneity. It is therefore recognized that there may be some variability between RPs and biosimilars. However, small variations between the reference product and the biosimilar have no significant impact on the quality and safety of the product due to the stringent development and approval process for biosimilars. Biosimilars undergo extensive comparability studies to demonstrate their high similarity to the reference product in terms of quality, efficacy, and safety. These studies encompass detailed analyses of the physical, chemical, and biological characteristics of the products, along with clinical trials to assess their efficacy and safety in humans [9,17].

It is relevant to clarify that a biosimilar is not a generic medicine in the definition of the generic term—the differences are presented in Table 1 [12,18]. Although biosimilars and generics are both variants of already approved, branded drugs, they have distinct characteristics and the terms should not be correlated [19]. Generics are chemically synthesized small molecules, which makes them chemically identical to RPs. In contrast, biosimilars are produced by a living cell. Even if two cells have the exact same sequence of amino acids in a protein, there is the possibility of natural variation in the glycosylation process or protein folding. In other words, generating a biological medicine equal to an RP is impossible. However, it is possible to produce a compound that has comparable excellence and physiological and scientific impacts, while ensuring its safety and effectiveness [19,20]. 

To establish biosimilarity, it is essential to evaluate whether the quality attributes (QAs) of a biosimilar and the RP are comparable. QAs are measurable product characteristics that describe the main properties of a drug molecule. Unlike generics, biologicals are molecules with intrinsic variability, which makes their QAs heterogeneous and susceptible to changes throughout the manufacturing process. It is important to conduct a comprehensive analysis of any differences that exist, to ensure that these are not clinically [23] relevant to the point of compromising the product’s function, immunogenicity, and efficacy. Therefore, unlike a generic, a biosimilar must present clinical and non-clinical data that prove its similarity, i.e., comparability studies must be carried out (this topic will be discussed in depth in Section 3) [17,20].

### 2.2. Intended Copies, Biobetters, and Standalone Biologics 

Biosimilars should not be confused with intended copies, biobetters, and standalone products, which are related but entirely different concepts [29]. Physicians are not required to recommend biosimilars solely due to their low cost. This decision should be based on scientific evidence and an understanding of the differences between them [30]. 

Intended copies are copies of an RP that do not align with the guidelines set forth by the EMA/FDA and WHO. Therefore, they are not accessible in heavily controlled markets, like the United States, Europe, and Australia, but are promoted in less regulated countries. Because they are cheaper, accessibility to biologicals in these countries has increased. For example, reditux and kikuzubam are intended copies of rituximab, available in India and some South American countries. The first one has undergone a phase III study to confirm its efficacy but has not undergone a direct comparison with the original rituximab. Kikuzubam was removed due to a lack of safety and confirmed toxicity. It has not been proven that intended copies offer the same effectiveness, quality, and level of security as the reference medicine. 

Even if the amino acid sequence is indeed the same, the pharmacological profile of the molecule can be affected, either by the existence of impurities, cluster development, or the occurrence of post-translational modifications (PTMs), for example. There is a lack of clinical trials to compare the effectiveness and safety of these medicines or to determine whether they are equivalent or non-inferior based on an acceptable number of patients. They are also not announced on global biosimilar news websites and do not have a registered protocol on clinicaltrials.gov (or it is not followed or not verified) [3,18,30].

Biobetters, in turn, are deliberately modified versions of other biological drugs, to improve certain attributes of their pharmacological profile, such as the dosage regimen, safety, efficacy, or immunogenicity [31,32]. Their manufacturing process is similar to that of biologicals; however, it involves using various advanced methods such as albumin replacement and pegylation (the addition of PEG), among others [31,32]. Biobetters are considered to be distinctive biological entities due to their unique molecular structures and functions. As a result, they must undergo the standard approval process prescribed by regulatory agencies, rather than the biosimilar approval process [19]. For example, insulin glargine is a biobetter that resulted from an alteration in the polypeptide chain of insulin; it was formulated to slow down the discharge of insulin molecules following subcutaneous administration [18]. 

Darbepoetin alpha is a modified version of epoetin, with an extended elimination half-life, due to alterations in glycosylation [18]. Another example is neulasta, a biobetter of neupogen, whose dosage frequency is once per cycle of chemotherapy, while neupogen is required once a day during the chemotherapy cycle. In addition, neulasta is more effective than neupogen, resulting in greater adherence. The lower dosage frequency, along with superior efficacy, means a lower economic load for each scheduled dose administration [18,30].

In July 2021, Sorrento Therapeutics (a pharmaceutical corporation committed to creating innovative drugs for infectious diseases, chronic pain, and cancer) granted approval for the marketing of the biobetter version of infliximab (CMAB008) in China. The utilization of Chinese hamster ovary (CHO) cell lines in production is anticipated to ensure greater safety and reduced immunogenicity, in comparison to the marketed tumor necrosis factor-α Ab, produced in murine cell lines [33].

Finally, standalone biologics are a novel class of medicines that are distinct from existing drugs. Their efficacy and safety are assessed by comparing them to a placebo or an appropriate comparator. In essence, these medications are the biological counterparts of me-too drugs and can be categorized as biosimilars [18]. 

## 3. Development and Regulatory Approval of Biosimilars

### 3.1. Development of a Biosimilar 

**Figure 1:**
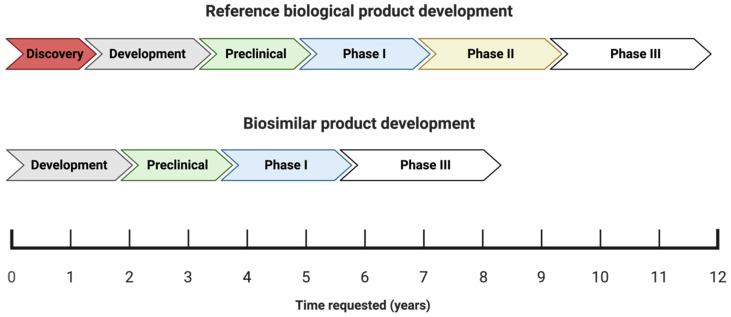
Schematic illustration of the development phases and the timeline (in years) of reference biologics versus biosimilars.

The process of developing and attaining approval for biosimilars differs significantly from that of new and innovative drugs (chemical or biological), as shown in Figure 1 [21].

The process of obtaining approval for new biological medicines typically spans a period of approximately twelve years, which involves a rigorous research and development phase aimed at creating a suitable molecule. The molecule is then subjected to thorough analysis during the preclinical stage, a critical phase in the drug development process [19]. The process of bringing a drug to market typically involves several established phases: I, II, III, and IV. Once a drug has been commercialized, phase IV begins. In the case of generic drugs, the process is simpler because the drug molecule has already been established and characterized. Therefore, all that is required is the production of the finished product and bioequivalence testing [18]. Since biosimilars are essentially copies of existing molecules with established product characteristics, there is no need to carry out the initial discovery or efficacy phase (phase II), thus shortening the development time to eight or fewer years and reducing the development costs by 10–20% [5,19,34]. Although Figure 1 shows that the development of bioismilars goes through a phase III, it is important to mention that phase III is not a clinical study as we know it, but rather a small clinical study whose main objective is to compare the efficacy and safety. A crucial point in the development of biosimilars is proving that the intended biosimilar exhibits similarity in both its analytical and biological functionalities when compared to the RP.

In other words, biosimilar medicine development lies somewhere between innovative drugs and generics. Although the molecule is established at the beginning of the process, as with generics, its reproduction and characterization are not an easy process, which constitutes a drawback [9,18]. Thus, biosimilar manufacturers face a huge challenge: the manufacturing process of the RP is proprietary, i.e., its details are not publicly available—biosimilar production, formulation, and administration are expected to be similar to those of the RP, without relying on the knowledge of the manufacturer in this case. In this way, the biosimilar manufacturer must extensively analyze the RP and use a method of “reverse engineering” in order to obtain a product with high similarity to the RP [8,18,35,36]. Healthcare professionals should understand therapeutic equivalence and “comparability” to provide the best care. Regarding generics, therapeutically equivalent medications are defined as having an identical chemical composition and the same pharmacokinetic profile. On the other hand, “comparability” is used in the context of biosimilars and means that comparable efficacy and safety have been verified between them and the RP (but this does not mean that they are identical, and it does not guarantee therapeutic equivalence) [37]. 

When a new biosimilar is designed, a well-established, step-by-step approach according to scientific principles and risk assessment must be employed [21]. Then, “quality-by-design” (QbD) constitutes an important tool, which allows the biosimilar to achieve higher similarity regarding the RP. It is essential to select an appropriate RP, obtain the reference active principle, identify the RP’s “quality target product profile” (QTPP) and critical quality attributes (CQAs), and develop a manufacturing process that allows the matching of the RP’s attributes. Thus, to accomplish this approach, characterization and matching of the QTPP is necessary [12,16,21]. 

First, the RP’s quality attributes (QAs) must be defined. It is crucial to measure the range of variation for quality attributes that directly affect the effectiveness or safety of the RP (CQAs). This can be achieved by using various batches of drugs to profile the quality of the proposed biosimilar’s target product [3,21,38]. In other words, CQAs are chemical, physical, biological, and microbiological characteristics that are defined, measured, and monitored continuously, to guide the clinical profile and establish clinical comparability [12,20,21]. For example, the CQAs of infliximab (which is the RP of the biosimilar CT-P13) are numerous and include those related to the structure, biological, function, content, and impurities [38]. The first step in the process is to choose the expression system, which includes the cell line and expression construct. This decision is critical given its potential to influence the translation and post-translational modifications, as well as to ascertain the quantity and nature of impurities or contaminants present in the final product [39]. While biosimilars are designed to mirror the polypeptide sequence of the RP, there is the possibility of detecting low-level sequence variations through the use of extremely sensitive methods. These variants can be derived from mutations in DNA or incorrect incorporation, resulting from a mistranslation or the improper acylation of the tRNA. In addition, biological products are subject to PTMs during cellular expression, including N- or C-terminal modifications such as amino acid cleavage, N-acetylation, methylation, and, most importantly, glycosylation (this affects biological function). The final quality of a product is impacted by a number of factors, including the methods used for purification, formulation, and storage, as well as the container closure systems used. RP manufacturers employ proprietary growth and purification conditions, as well as cell lines that are especially adapted for their processes. For biosimilar manufacturers, simply having knowledge of the protein sequence or the cells used is not enough to create an identical biological product. Therefore, minimizing the structural differences between the biosimilar and the RP is crucial, as even minor variations could potentially impact the product’s PK, efficacy, safety, and immunogenicity [21]. 

#### Post-Translational Modifications (PTMs)

Several factors may affect the similarity between the suggested biosimilar and the RP, and PTMs are a huge challenge for the pharmaceutical industry. 

mAbs are subject to various modifications throughout their production, purification, and storage processes, resulting in different forms. The genetic sequence dictates the amino acid arrangement in a protein, but its structure, stability, and function will be determined by the PTMs.

Proteins can undergo PTMs through the addition of functional groups, cleavage, or degradation. Recombinant mAbs often experience PTMs, such as N- and C-terminal modifications, deamination, glycosylation, glycation, phosphorylation, acetylation, sulfation, alkylation, methylation, proteolysis, oxidation, mismatched S-S bridges, and truncation. Among these, glycosylation has the most significant impact on biological function [40]. 

Proteins undergo glycosylation, in which carbohydrate fractions are added during polypeptide synthesis or in the endoplasmic reticulum and Golgi apparatus of the cell. Proteins undergo two primary forms of glycosylation: O-linked and N-linked. O-linked glycosylation involves the addition of glycans to serine or threonine amino acid residues through an oxygen atom. On the other hand, N-linked glycosylation initiates with the attachment of a high-mannose-based structure to an asparagine amino acid residue within an Asn-X-Ser/Thr consensus sequence during the process of translation. The modification occurs downstream in the endoplasmic reticulum and Golgi apparatus, and X can represent any amino acid except proline (Pro) [40]. 

The prevailing form of glycosylation observed in monoclonal antibody (mAb) therapeutics is N-linked oligosaccharides [40,41]. The N-glycosylation of therapeutic antibodies involves PTMs affecting biological function and treatment effectiveness. The characterization of these glycans is required by manufacturers and regulatory agencies, but their complex structure and heterogeneity present challenges [40].

Extensive analytical testing platforms are necessary to verify the protein’s identity, maintain a consistent manufacturing process, and ensure product quality. A single error in modifying or combining components could lead to adverse reactions in patients or a reduction in the effectiveness of the final product. Liquid chromatography (LC), mass spectrometry (MS), electrophoresis, and spectroscopy serve as effective tools in confirming the biological identity, sequence variations, and glycosylation patterns, as well as other PTMs and impurities, in antibody products [13,41,42]. 

Peptide mapping is a technique used for the analysis of a biopharmaceutical’s primary structure and it is recognized by the International Council of Harmonization guidelines (ICHQ6B). Peptide mapping assumes a crucial role within the biopharmaceutical industry, serving to authenticate the identity of a protein therapeutic and monitor degradation events like oxidation or deamination. It offers a comprehensive analysis of the protein under scrutiny, making it a standard method for the characterization of mAbs [41,42,43]. In this way, it is crucial to thoroughly examine any disparities in PTMs to safeguard the safety and efficacy of the proposed biosimilar [13]. 

The target protein is digested with a specific enzyme to sequence specific cleavage sites. Thus, because of digestion, peptides with an ideal molecular weight for LC/MS analysis appear, and it is then possible to determine the molecular mass accurately. If we use the MS/MS (MSE) analysis method, we can check a large percentage of the sequence. If we combine one or more enzymes, we can achieve 100% coverage of the sequence. This comprehensive approach not only facilitates the identification of modifications but also reveals their specific locations within the protein sequence [41,42,43,44,45].

However, it should be taken into account that, as this technique involves sample preparation steps, it is subject to variations due to the differences between techniques, technicians, and/or laboratories. Differences in product batches or biosimilar products can make it difficult to compare and reproduce results over time, potentially affecting the quality, efficacy, and safety of the product. For this reason, the sample preparation conditions should be promoted to minimize the degree of artificially induced modification [44]. 

However, the conditions responsive to effective reduction, alkylation, and digestion often coincide with those that trigger unfavorable alterations [42,44]. 

Several methods that automate the digestion process and reduce the time required to prepare samples for peptide mapping are already available on the market. These methods provide significant improvements in reproducibility, which leads to fewer failures and the easier interpretation of data. In order to accurately characterize the protein, it is essential to have a comprehensive understanding of its distinctive traits and qualities, which will aid in the creation of a precise peptide map [13,41]. 

### 3.2. How to Build the Evidence for Biosimilarity

The purpose of the biosimilar investigation pathway is to show that the proposed biosimilar has a safety and efficacy profile that is neither better or worse than that of the RP, rather than to demonstrate superiority or inferiority between the two [9]. The demonstration of biosimilarity is achieved through a step-by-step approach, as approved by the EMA and the FDA: first, a study of physicochemical and biological comparability (quality studies) is carried out, followed by non-clinical comparability (in vitro and in vivo studies) and, finally, clinical comparability [4,12,15,46,47]. The clinical comparability study is generally conducted in successive steps, starting with PK and, if possible, PD studies, followed by at least one clinical trial of efficacy and tolerance [4]. However, in most cases, quality studies performed in vitro are sufficient to confirm that the modifications are insignificant from a clinical perspective. It is important to note that the two products do not need to be identical; it is only necessary to show that there is no clinically significant change (i.e., the proposed biosimilar and the RP need to be comparable) [18].

**Figure 2:**
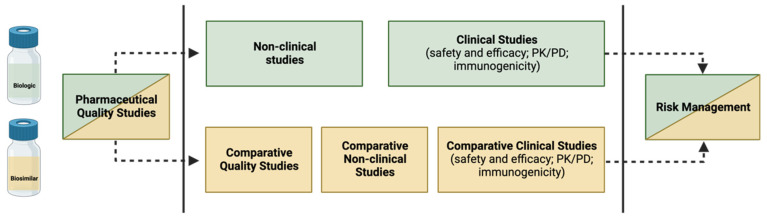
Building evidence for biosimilarity—comparison of data requirements for approval of a biosimilar versus an RP.

The (bio)physical characteristics of a biosimilar are heavily influenced by each step of its development process. Therefore, it is imperative that no step refutes or overcomes substantial distinctions encountered in the preceding steps, and that all three stages demonstrate adequacy to affirm the biosimilarity. Despite the potential depth of biosimilar development comparable to that of an RP, the primary focus lies in achieving comparability during the initial phases of development. In contrast, the focus for a new biological agent is on establishing clinical efficacy and tolerability [18]—as shown in Figure 2.

#### 3.2.1. Demonstration of Analytical Similarity—Comparative Quality Studies

Analytical assessments constitute a repetitive and iterative process, whose aim is to evaluate the standard of the suggested biosimilar in comparison to the RP. Creating biosimilars demands substantial effort, with a particular emphasis on assays that offer a sensitive assessment of similarity. To ensure comparability in quality, extensive analytical characterization, receptor binding studies, and bioassays are necessary to confirm that the molecular structure and functionality are alike. These studies are carried out through in vitro assays, which are sensitive techniques capable of detecting distinct clinical characteristics between the biosimilar and the RP [12,16]. Although there may be minor variances in structure, such as glycosylation distinctions, the product can still be classified as a biosimilar as long as these variances do not significantly affect its safety or efficacy. To guarantee that the biosimilar closely resembles the RP, it is crucial to test multiple batches over a defined period and establish a thorough QTPP [12,21]. 

Quality attributes (QAs) are compared using analytical methods such as surface plasmon resonance (SPR), enzyme-linked immunosorbent assays (ELISA), mass spectrometry, and flow cytometry. The QAs to be evaluated include the physicochemical properties, biological activity, immunochemical properties, purity and impurities, quantity, strength, thermal stability profiles, and other modifications, such as oxidation and deamination [12,16,21].

The PK comparison involves evaluating the PK parameters, as well as analyzing the composition, physical properties, primary structures (amino acid sequence and disulfide bond), and higher-order structures (e.g., local and three-dimensional conformation) of the biosimilar. As mentioned above, it is important to verify that the amino acid sequence matches the RP and to compare the N- and C-terminal amino acid sequences, free SH groups, and disulfide bonds. If there are PTMs, such as glycosylation, oxidation, and deamination, these must also be defined. If their presence is verified, it is necessary to compare the carbohydrate structures, including the glycan profiles and glycosylation patterns. The assessment of the biological effects relies on the characteristics of the substance and generally comprises mAb–antigen binding, Fcγ receptor binding, and FcRn binding [12]. The characterization of the purity and impurities related to the product and its manufacturing process helps to guarantee its safety, and they must be determined and compared qualitatively and quantitatively, through a combination of analytical procedures [21]. To ensure product quality and safety when working with biological sources, it is important to consider their shelf life and characteristics. Understanding these factors helps to make informed decisions about utilization and storage. All process-related impurities must be determined (e.g., host cell DNA and proteins, reagents, downstream impurities, etc.), as well as their potential risks (e.g., immunogenicity) [16]. The thermal stability assessment evaluates forced degradation profiles and degradation products [21]. 

**Figure 3:**
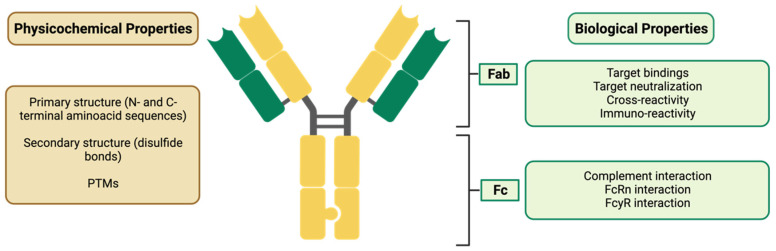
Characterization of a biosimilar mAb, evidencing its physicochemical and biological properties. Fab: fragment antigen binding; Fc: fragment crystallizable; FcRn: neonatal Fc receptor; FcγR: Fc-gamma receptor.

In the case of biosimilar mAbs (for which some of their QAs are represented in Figure 3), assessments of the biological activity to bind to the Ag, the connection to the Fcg receiver, the FcRn binding, the antibody-dependent cellular cytotoxicity (ADCC), and the complement-dependent cytotoxicity (CDC) must also be carried out. In addition, the affinity and Ag binding specificity of the biosimilar and the RP must be compared. 

When considering the efficacy and safety of monoclonal antibodies (mAbs), the importance of differences in biological activity related to the Fc regions will vary depending on the mAb’s mechanism of action. If the mAb exerts ADCC activity, it is important to carefully consider the difference in FcgRIIIa binding and ADCC activity. On the other hand, if the mAb’s mode of action does not include ADCC activity (e.g., an mAb against soluble monomer Ag), it is possible that the difference in binding will not significantly impact the mAb’s efficacy and safety [16]. 

The folding and conformation of the CH2 domain (Abs are flexible macromolecules consisting of two identical light chains and two heavy chains, also identical, alloys through dissulfide bridges. The heavy chains have a variable domain (VH), with great diversification in their aminoacid composition and three constant domains (CH1, CH2 and CH3), which present homogeneous amino acid sequences responsible for the effector actions of immunoglobulins (CH2 and CH3). The light chains are formed by a constante domain (CL) and a variable domain (VL). The CH2 and CH3 domains the Fc region (crystalline fragment–effector), while VH, VL, CH1 and CL constitute the Fab) within the Fc region of the IgGs depends on the N-glycans attached. Glycosylation causes the CH2 domains of IgGs to have higher stability, unlike deglycosylation, which causes instability, making them more prone to unfolding and disaggregation. It is also known that N-glycosylation has an impact on ADCC and CDC activity, through the modulation of the connection to the Fcγ receiver. Therefore, N-glycosylation can alter the therapeutic potential of mAbs, in a clinically significant way, and is considered a CQA [40,42].

#### 3.2.2. Establishing Non-Clinical Biosimilarity

The purpose of the non-clinical comparability exercise is to evaluate how alike the biosimilar and the RP are in regard to their mechanisms of action, their functional activity, and their quality characteristics [48]. Physicochemical and laboratory analyses are conducted in vitro during PD studies in which ligands bind to the physiological targets and the physiological effects on cells are evaluated. They may activate or inhibit the receptor; thus, the cell function may increase or decrease. The preclinical comparison of the PK and PD can help to minimize residual uncertainty regarding their similarity [21]. In vivo PD studies are only used if there are no in vitro models that meet the required parameters [15,46]. 

Evidence from animal studies (in vivo studies) continues to be a controversial and sensitive issue, and guidelines recommend that their use should be minimized or eliminated wherever possible, by implementing, for example, the 3Rs (Replacement, Reduction, and Refinement). It may be necessary to perform additional in vivo studies, from a non-clinical perspective, to complement the data obtained during the analytical step of biosimilar development. One reason for conducting these studies could be to explore any relevant discrepancies in the properties of the biosimilar and the RP that were not identified during the initial analysis, such as differences in the composition or the use of less common excipients in the formulation [12].

The need for an in vivo assessment will determine the focus of the study, as more information must be required. Such an assessment might involve a quantitative analysis of the PK and PD profiles of the biosimilar and RP, which includes a concentration–response dose comparison. In the case of safety studies, and if simians are considered the only applicable species, a flexible approach should be considered. Toxicity studies and standard repeat-dose toxicity studies may be applied when the production of a biosimilar occurs in a new organism or cell and when the presence of excipients is verified. Nevertheless, these studies should not be performed in non-human primates if not absolutely necessary. Quantitative and qualitative alterations in product-related variants (e.g., glycosylation), which may cause hypersensitivity, should be clinically evaluated [12,16,21]. Although animal immunogenicity studies cannot predict immunogenicity in humans, it is possible to collect blood samples from animals for the further assessment of PK/toxicokinetic data. Safety pharmacology, reproductive toxicology, and carcinogenicity studies are not mandatory. Typically, local tolerance studies are not necessary, but if there is insufficient information on the route of administration for certain excipients, they may need to be reviewed [12,21].

#### 3.2.3. Clinical Considerations—The Supporting Role of Phase I and Phase III Clinical Studies

**Figure 4:**
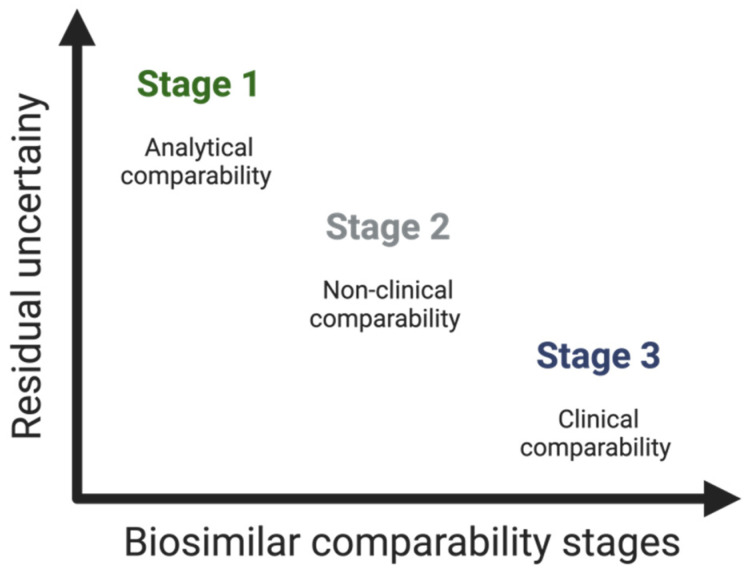
Residual uncertainty along the stages of the comparability study of biosimilars.

Clinical studies constitute the third stage of the comparability study [49]. As mentioned, the establishment of biosimilarity focuses, above all, on preclinical aspects and, particularly, on the quality of the biosimilar. Thus, the number and scope of clinical studies executed rely on the level of uncertainty regarding biosimilarity, determined by previous analytical assessments (and non-clinical in vivo testing, if executed)—see Figure 4 [12,21]. 

Briefly, in this third step of the comparability study, the aim is, through the analysis of analytical data or previous studies, to rule out clinically relevant PK/PD, clinical safety (immunogenicity), efficacy, extrapolation, and PV differences—and thus confirm the biosimilarity of the proposed biosimilar in relation to the RP [20,21,24]. In other words, while reference biological medicines are evaluated in controlled trials to demonstrate their clinical benefit, biosimilar clinical trials are mainly responsible for demonstrating the clinical equivalence between the potential biosimilar and RP [16,17]. 

In this context, one notable instance is the clinical study conducted for the proposal of the first biosimilar of natalizumab (NTZ), for patients with relapsing–remitting multiple sclerosis (RRMS). This biosimilar was developed by Polpharma Biologics SA as an alternative to the reference medication natalizumab (ref-NTZ), in accordance with the guidelines of the US Food and Drug Administration (FDA) and European Medicines Agency (EMA). The study was a randomized, double-blind phase III trial, involving 264 adult patients with RRMS, who were treated with either biosim-NTZ or ref-NTZ. The results demonstrated that biosim-NTZ provided comparable efficacy, safety, and immunogenicity to ref-NTZ, with a similar incidence of new active lesions observed in both treated groups. This study enabled concrete data to be provided to the regulatory authorities, supporting the proposal of biosim-NTZ as a biosimilar alternative to ref-NTZ for the treatment of RRMS. This, in turn, allowed for a significant reduction in costs and increased patient access to treatment [50].

##### Pharmacokinetic and Pharmacodynamic Studies

The process of developing a biosimilar typically starts with a study that aims to prove that the proposed biosimilar is similar in terms of its PK and PD properties when compared to the RP [21]. The study design depends on several factors, namely the clinical context, safety, and PK of the RP. Therefore, it is only carried out after being extensively characterized [12,16]. 

PK assessments are needed to compare the biodisponibility of the drug, which includes absorption, disposition, time dependence, and binding to blood components. PD studies, in turn, ensure that the biosimilar’s efficacy in the target tissue is equivalent to that of the RP and that the mechanism of action is identical. In some cases, comparative PK/PD studies might be enough to show that the clinical outcomes are similar [12]. 

These studies must be carried out in an appropriate population, and, whenever possible, it is preferable to use only healthy individuals, as this guarantees a homogeneous population, composed of immunocompetent subjects who are not receiving any concomitant medication [21]. In most cases, a single-dose study is sufficient to evaluate absorption and compare different administration techniques. However, it is crucial to consider that soluble receptors have the potential to interact with the therapeutic protein, thus affecting the PK profile by affecting the clearance or volume. Furthermore, it is important to investigate any possible binding to plasma proteins like albumin and acid α-glycoprotein. When performing these PK assessments, several factors need to be considered. This includes possible chemical changes in proteins, individual differences, drug interactions, and populations with specific pathologies (e.g., renal or hepatic impairment) [12]. 

##### Efficacy Studies

Based on the current regulatory guidelines, certain situations do not require comparative clinical efficacy studies. The FDA states that a comparative clinical study is necessary “if there is residual uncertainty about whether there are clinically meaningful differences” between the proposed biosimilar and the RP, “based on structural and functional characterization, animal testing, human PK and PD data, and clinical immunogenicity assessment” [47]. The type and amount of clinical data needed depend on the complexity of the therapeutic mechanism, as well as the availability of an endpoint that correlates with effectiveness. The EMA has waived the need for rigorous comparative efficacy, safety, and immunogenicity studies in certain situations, while still emphasizing the importance of comparative PK/PD studies [15,46]. 

Efficacy studies make it possible to analyze the significant differences that exist in terms of treatment efficacy, i.e., their main goal is not necessarily to prove effectiveness, but rather to confirm that the clinical performance is comparable [12]. These studies require randomized parallel-group comparative clinical trials (preferably double-blind trials), as well as appropriate efficacy endpoints. The detection of potential differences related to a product should be sensitive enough to ensure that any impact caused by individual- or disease-related factors is decreased. In order to ensure a high-sensitivity study, the population chosen should closely resemble the one given in the approved indication for the RP. This will enable the detection of differences between the proposed biosimilar and RP regarding efficacy. Other factors, such as prior treatments, concurrent medications, and disease severity, must also be taken into account to provide the maximum sensitivity [16,51]. 

##### Safety Evaluation 

The safety issues related to the biosimilar play a major role in comparability studies. According to the usual procedures in the development of biological medicines, the biosimilar’s safety profile is built across the entire clinical program—during phase I PK/PD studies and phase III direct comparison studies [12]. 

To establish the similarity between a biosimilar and its RP, it is necessary to assess and compare the type, severity, and frequency of any adverse events (AEs) that may occur. Additionally, any potential safety risks arising from variations in the manufacturing process must be taken into consideration [12]. Moreover, immunogenicity must also be intensively studied, due to the possible immunogenic character of biologicals. The length of the immunogenicity study should be rationalized based on individual cases, since it relies on factors such as the duration of the treatment, drug release, and the time that it takes for the immune response to manifest. If there is an increase in the immunogenic profile of the biosimilar in relation to the RP, this can become a problem for the risk–benefit analysis (this does not occur if the immunogenic profile is lower in the biosimilar) [4,12]. 

### 3.3. Regulatory Concerns

In the last fifteen years, the EMA and/or the FDA have given their approval to approximately one hundred biosimilars, and this number is predicted to grow even more in the next years. For their approval, it is necessary to ensure robust regulation [19,20]. Regulatory bodies and manufacturers are mainly responsible for guaranteeing that biosimilars intended for use closely resemble the RP in both structure and function. This is essential to prevent any adverse effects on the efficacy and safety of the biosimilar. By means of thorough evaluation and demonstration, any structural discrepancies that may have an impact on the clinical outcomes must be eliminated [9].

Biosimilars have very particular and exclusive approval conditions, and implementing an abbreviated licensing pathway can present several challenges [21]. Therefore, the EMA and the FDA have developed specific and consistent regulatory guidelines that must be followed to approve the final product—the EMA’s guidelines are presented in Table 2 [17,46]. However, divergences exist between the regulatory agencies, so the number of biosimilars approved in the various markets is very different [8]. The EMA was the pioneer among regulatory agencies in establishing a system for the approval of biosimilars in 2003. Then, in 2006, the first biosimilar (Ominotrope^®^) was approved [15]. Almost a decade later, the EMA was followed by the FDA, which adopted the same principles and approved the first biosimilar in 2015 [15]. Both agencies recognized the complexity of developing biosimilars, highlighting the fact that each biosimilar candidate has distinct and particular characteristics, making it crucial to design targeted development programs on a case-by-case basis. The WHO has made recommendations to promote global growth regarding these products, requiring at least the existence of clinically relevant parameters, including PK, PD, safety, efficacy, and immunogenicity data, to consider biosimilarity [12,21,52]. In cases where substantial evidence of similarity is verified in all preclinical data, it may not be necessary to conduct efficacy studies. However, if any differences are noted, further toxicological and/or clinical investigations are required to address any remaining uncertainties and potential immunogenicity concerns [8,31]. 

US and EU laws state that an RP must be approved according to the legislation established by local authorities. While, in the US, the proposed biosimilar must demonstrate its similarity to the RP approved there in order to be accepted, for EU approval, it must be similar to the RP with approval in the European Economic Area (EEA) [5,21]. These regulatory agencies have implemented measures to allow the use of foreign comparators in comparative clinical studies, if scientifically justified, due to the complexity and cost involved in developing biosimilars. From a regulatory and legislative perspective, the acceptability of reliance on clinical data generated using a comparator of foreign origin depends on the successful establishment of the “scientific bridge”. The “scientific bridge” between the product of local origin and that of foreign origin must consist of a comprehensive assessment of the analytical similarity of the proposed biosimilar with both comparators. In addition, a three-arm PK similarity analysis must be conducted. This analysis should confirm the bioequivalence between both comparator arms and also the candidate biosimilar in contrast to the respective comparators [21,31]. 

Another regulatory consideration, exclusive to the US, is the determination of interchangeability. Under US guidelines, an interchangeable biosimilar is a product that is biosimilar to the RP, and it is hypothesized that the intended therapeutic result will be attained in all individuals [53]. When the RP is substituted with a biosimilar, the risk regarding efficacy and safety cannot be greater than if there is no substitution. Prerequisites for interchangeability are not required in the EU regulatory framework [15]. Although the EMA is mainly responsible for the approval of biosimilars, each member state (MS) has its own regulations [21,31]. 

To ensure the successful acceptance of biosimilars, it is important to establish clear naming criteria and effective safety monitoring. In this regard, to achieve unique names, a four-letter suffix is added to the international nonproprietary name (INN). This helps to prevent misconceptions among pharmacists and ensures that adverse events are accurately attributed to the correct manufacturer. Proper PV is essential for the safe and effective use of biosimilars [21,31]. 

These issues of interchangeability, substitution, extrapolation, and nomenclature will be explored in Section 5.

## 4. Post-Marketing Monitoring of Safety of Biosimilars—Pharmacovigilance

PV, in accordance with Good Pharmaceutical Practices, aims to identify, quantify, evaluate, and avoid the potential threats linked to the utilization of commercialized products, with the aim of improving the safety of medicines, in defense of users and public health [7,24]. According to the literature, clinical studies are usually insufficient to identify rare AEs and, in addition, their clinical development program is poorly suited to the identification of tolerability risks, as it is known to be shorter than the RP’s. Thus, in clinical terms, product tolerability must continue to be monitored during the post-marketing phase [4,9,21,37]—it is especially significant for these medicines since their protection is affected by the ability to produce an immune response, adverse reactions caused by hypersensitivity, a greater possibility of experiencing additional adverse events, and because of their vulnerability to variations in the manufacturing process [7,37,54]. The manufacturer needs to have a PV system in place that can detect, evaluate, and prevent the appearance of a drug-related AE during its manufacturing. Thus, PV systems should not simply track the types and severities of AEs to identify new class-based risks but should also be robust enough to detect the number of times that AEs occur over time [7,8,24]. As part of the approval procedure, the candidate is required to provide a summary of the initiative, as well as a risk mitigation strategy that adheres to current European Union regulations and pharmacovigilance recommendations. 

It is important to ensure that any monitoring requirements placed on the RP are properly accounted for in the biosimilar’s pharmaceutical co-surveillance plan, and immunogenicity must also be considered in this context [4].

After the product is introduced into the market, the EMA has well-established PV programs for the monitoring of AEs. The network established by the EMA to report and evaluate suspected adverse events during development and after marketing authorization is known as EudraVigilance. Information on drug safety is collected through spontaneous reports by healthcare professionals and patients [8]. In order to improve the results, it is preferred that the reports provide a thorough and detailed analysis, including the nature of the AE and information on the drug (e.g., proprietary name, INN, lot number, and dosage given). However, correct causality can be difficult to assess, since patients who are undergoing therapy with biopharmaceuticals are often in polytherapy and are individuals with serious diseases and/or risks to life. AEs are often underreported or have incomplete reports, with varying rules for reporting across different countries [8,37]. 

In all medicines, the Summary of Product Characteristics (SmPC) and the Information Leaflet (IF) must include a text urging individuals to report any suspected AE, through national spontaneous reporting systems and/or official forms available on the internet [24]. In the meantime, a new concept has been introduced by the latest EU PV legislation, where a list of drugs subject to additional monitoring during a certain period has been released. For this, these drugs are identified with a black symbol (inverted triangle) with corresponding text in the SmPC and IF. With this new approach, the strengthening of the PV of all medicines has been seen, increasing the transparency, communication, and trust [55,56].

Since the initial launch of the first biosimilar in Europe over fifteen years ago, there have been no notable variances in the safety profiles of these products. However, their monitoring remains extremely important, particularly in pediatrics, as the risks and comorbidity profiles may differ between children and adults [8].

## 5. Controversies in the Use of Biosimilars

Despite the numerous benefits that biosimilars bring, there exist notable disparities in their utilization, namely cost savings and market shares. These disparities pose significant questions regarding the safety, efficacy, and interchangeability of these drugs [56,57].

### 5.1. Immunogenicity 

Immunogenicity is the ability of a specific substance to trigger an adverse reaction as a result of multiple factors [48]. This immune response is complex and, in addition to the formation of Abs, it also involves other events, such as the activation of T cells or the activation of the innate immune response [35,48]. The immunological response’s characteristics are a crucial component regarding regulatory clearance for biosimilars, i.e., an essential component to prove the similarity of the biosimilar, in comparison to the RP, being evaluated through rigorous quality, non-clinical, and clinical studies [3,4,25,35,48,51,55]. 

As previously mentioned, biological proteins have a high molecular weight and a very complex composition. For this reason, when these drugs are administered to patients, they can produce undesired immune responses, stimulating the formation of anti-drug antibodies (ADAs), which may cause immune-mediated toxicity (retarded hypersensitivity and anaphylactic reactions) or compromise the effectiveness of the treatment [25,55]. However, immunogenicity alone does not translate into safety, since this adverse reaction is very rare and, in most cases, the immunological reaction is not even related to the clinical consequences (e.g., ADAs can be transient) [3,15,46,55,58]. Moreover, the nature of the reaction is related to several factors, such as alterations in product development, the stability characteristics, and the protein structure during storage; treatment-related factors (risk is related to how it is administered—subcutaneously or intravenously); and the patient/disease factors (e.g., age, immune system status, genetic history, concomitant medication) [4,32,55,57]. It is also important to mention that it is very unlikely that harmful immune reactions will occur once changes have occurred in the manufacturing process or after switching from one biological to another, as the comparability studies show that the batch received has comparable standards and is devoid of any contaminants that may lead to an immune response [15,35,47]. Finally, another very important aspect is that immunogenicity is always subject to post-authorization monitoring, which must be guaranteed by manufacturers, pharmacists, and physicians [26]. This step is extremely important in detecting rare but harmful immune responses that can only be detected after a prolonged monitoring period of multiple patients—therefore, the immunogenicity assessment must be part of the post-authorization risk management plans (RMP) and PV activities [15,26,35,47,48]. 

The EMA has published useful guidelines on immunogenicity; however, as each product has specific considerations, finding an analogous method for various biological substances presents a challenging task, so each manufacturer must justify the method used to evaluate it [1,46]. 

### 5.2. Extrapolation

Biosimilars can incorporate the clinical indications of the RP without conducting clinical trials for the same indications, thanks to extrapolation, a key concept in their development and approval [56,58,59].

However, for extrapolation to be deemed valid, it is crucial that the data used are derived from studies using a highly sensitive clinical model to identify any potential disparities in safety, efficacy, or immunogenicity between the RP and the potential biosimilar. The group of individuals included in this sensitive study may consist of patients who differ from those involved in the crucial clinical trials of the RP. PD measures are regarded as delicate clinical endpoints and might be chosen as the primary objectives of the biosimilar clinical trial [5,8,21,49]. Moreover, to be considered supportive for extrapolation, it is important that the indications follow the same molecular mechanism, with the same receptors and dosage response, and activate the same molecular pathways when they bind to their targets, while also being located and expressed similarly [5]. The complete dataset should include detailed PK and biodistribution data, coupled with sufficient information pertaining to safety and immunogenicity. This meticulous approach is imperative to ascertain that the biosimilar does not introduce any heightened safety concerns when juxtaposed with the RP [5,38,47]. 

This approach has been the subject of immense criticism and has been discouraged by several professional medical societies [52,55]. Healthcare professionals may express reservations about recommending a biosimilar for off-label purposes that has not undergone rigorous clinical evaluation, despite the fact that the RP has been authorized for these particular uses. However, since extrapolation is based not only on clinical, but also on structural, physicochemical, functional, and non-clinical data, this point of view must be clarified [1,5,48,52,55].

Regulatory agencies are mainly responsible for deciding whether to allow the applicability of data for different indications. Therefore, if enough scientific evidence is verified, with proof of biosimilarity and a known mechanism of action, extrapolation is approved by the EMA and FDA guidelines [52,53]. Regarding infliximab, the EMA and other regulatory agencies approved it for all of the RP’s indications, while others did not [12,55]. 

In accordance with the EMA-approved regulations for reference biological drugs, all data that come from clinical development are contained in the SmPC [52]. Thus, extrapolation between the RP and biosimilar is allowed, as long as it is supported by scientific evidence and evaluated through the analysis of all analytical, clinical, and non-clinical data [12]. However, the expansion or restriction of indications should not be accepted, and non-approved indications are considered off-label [5,35,48,55].

### 5.3. Interchangeability and Substitution

Once a biosimilar is authorized by a regulatory agency, it can be prescribed with guarantees of efficacy and safety, for all indications authorized in the SmPC [57]. For this reason, a physician, when starting treatment on a patient, can introduce an RP or a biosimilar [48,60] However, important questions emerge: can physicians replace the RP (which is being administered) with a biosimilar? Can the RP and biosimilar be used interchangeably with one another? To answer these questions, it is important to understand the terms “substitution” and “interchangeability”, which, despite being very similar, are quite different concepts and should not, therefore, be confused [5,19,55,56,59]. 

Substitution is the act by which the pharmacist replaces a drug with a similar one, without the need for the physician’s consent or the patient’s approval, since there is confirmation that the repeated exchange between these two drugs does not constitute an additional risk to its safety and does not reduce the effectiveness of the therapy [5,55,56]. Automatic substitution is applicable for most generics and plays a major role regarding economic factors. However, this method may not be suitable in some cases, since it could potentially affect the safety and PV programs. This happens, for example, with modified-release theophylline and calcium channel blockers—in these drugs, the difference between the therapeutic and the toxic effect is minimal. Therefore, it cannot be ensured that the biosimilar will have the same risk–benefit ratio [21,24]. Certain US states and European nations have implemented “do not substitute” lists, in order to prevent the automatic substitution of drugs. On the other hand, some European countries rely on the knowledge and experience of healthcare professionals to avoid incorrect substitution [1,46]. Distribution systems may be responsible for possible improper substitution if it is automatically allowed, and potential risks are not recognized by healthcare professionals [1,46]. Automatic substitution is not suitable for biopharmaceutical products. As previously stated, biosimilars are not identical copies of innovator products and even small variations could impact the clinical results [5,9,16,19,38]. Moreover, if automatic substitution is permitted, patients receive various biopharmaceutical products throughout their therapy, which makes it difficult to collect PV data: if an AE results from the use of different products without proper documentation, it becomes challenging to identify the specific product responsible during safety studies. This can result in the attribution of the AE to the incorrect product. Therefore, providers must have complete information on the biopharmaceutical product that their patients are using, to avoid such scenarios [5,18,24].

Regarding the term “interchangeability”, in the US, interchangeability is the designation that allows automatic substitution if the State Laws permit it. In the EU, interchangeability is the exchange of a medicine for another, which can either be carried out through switching (physician decision) or substitution (carried out by pharmacists) [5,12,55,56,59]. 

In all these cases, it is important to highlight the physician’s role as the last link in the decision chain when choosing the most appropriate drug for the patient. However, for the physician to choose and prescribe the most appropriate treatment, the regulatory bodies must position themselves regarding interchangeability and establish guidelines that detail when and how the exchange can be carried out. Nonetheless, at this point, there is great uncertainty, since the opinions of regulatory agencies around the world are heterogeneous, and the interchangeability and substitution of biosimilars are not characterized in detail—this is because biosimilars are not interchangeable per se [5,55]. According to regulatory agencies and scientific corporations, biosimilars may be recommended for patients who have not received biological treatment before (“primary naive patients”), those who were treated and then experienced a wash-out period, or those who have undergone successful treatment with biologicals for a chronic illness [5,8,55].

The FDA was the first regulatory agency to legally define interchangeability (in January 2017) [52,53]. In order to ensure an interchangeable status, the FDA requests the implementation of studies that demonstrate biosimilarity, as well as pre-marketing studies that address the multiple and reverse exchange of biosimilars and RPs [47,52,53]. Furthermore, clinical trials of PK and PD must be validated at the beginning of development. Therefore, according to the FDA legislation, a biosimilar is considered interchangeable with the RP when the data presented show that no increased risks related to safety or efficacy are verified [47,53,59]. When a biosimilar is considered interchangeable with an RP, automatic substitution is allowed by the FDA and the authorization of the prescribing professional is not required. 

Currently, the FDA has only granted approval for three biosimilars, considered interchangeable: adalimumab (Cyltezol^®^), insulin glargine-yfgn (Semglee^®^), and ranibizumab-eqrn (Cimerli^®^) [5,8,17,52,59].

Contrary to what occurs in the US, in the EU, each country is free to decide whether a substitution is allowed or not, because the EMA cannot classify a biosimilar as interchangeable across all member states of the EU [52]. However, recently, on 19 September 2022, it was announced that the EMA, on July 22, stated that EU-approved biosimilars would be interchangeable with their respective RPs or equivalent biosimilars. The interchangeability of biosimilars was already applied in several MSs, so this decision made the EU approach uniform. In this way, the process becomes simpler and clearer for healthcare professionals and allows more patients to use biologicals in the EU. The EMA’s decision was based on the experience of recent years, as it has become increasingly common for physicians to make these exchanges, without any associated problems (over the years, the use of biosimilars has been analyzed in more than one million patients and no safety problem has occurred) [61].

As regards decisions on substitution in the context of a pharmacy (dispensing without consulting the prescriber), it remains the decision of each MS [61]. 

### 5.4. Nomenclature

In the post-marketing period, it is essential to ensure a precise prescription and to avoid confusion between the biosimilar and its RP (as well as between the biosimilar and other biosimilars), so specific nomenclature is necessary [4,9]. This nomenclature will be extremely important to monitor the use of the drug throughout its life cycle, allowing us to report and track adverse events [55,58,62].

Organic products are so complex that it is impossible to use short, usable, and descriptive names. Their definition is very difficult and subjective—this is because the same criteria are not used to define unique products [4,58,62]. Active substances can be named based on their structures, approvals, and/or how they are marketed. However, this is not the case with biopharmaceuticals, as their naming involves identifying changes to the existing product. Thus, given the uniqueness and novelty of the product, a new name is required [4]. However, there are several different opinions: some consider that, as this product has an innovative formulation, a revised manufacturing process, new authorization, and a distinct trade name, among other reasons, it is considered a “new” product and, therefore, it requires a new name; others, on the contrary, consider these products to be similar enough to keep the same name. In contrast to all the studies involved in proving the innovative characteristics of a new product, biosimilarity and molecule authorization are not notably difficult [4,62].

The WHO collaborates closely with INN experts to select a single, globally acceptable name for each active substance to be marketed as a pharmaceutical. However, to achieve the intended objective in the post-marketing of biologicals and biosimilars, INNs cannot be used as the only means of biological identification. INNs work well for generic drugs, but not for biopharmaceuticals, as, in this case, it is important to recognize that even similar products should be viewed as distinct from one another [48]. In order to simplify the identification of the AEs of a product using the INN, the brand name should be used. Alternatively, a combination of the INN and a unique identifier, like a Greek letter or several letters, when referring to biopharmaceuticals, is also a suitable option [18,55,63]. 

Nevertheless, in specific situations, the same name is applied to different products. In this regard, “interferon beta-1α” is used to describe several pharmaceutical products. In other words, considering the increase in biosimilar development, INNs are becoming increasingly irrelevant [18,62]. Another alternative has thus emerged, proposed by the WHO, which consists of using a biological qualifier (a four-digit code) to distinguish biosimilars from RPs [18,52,55,63].

Some regulatory bodies suggest that the INN system should not be used to prescribe biosimilars. In 2012, the European Commission published Directive 2012/52/EU, establishing that the use of brand names is mandatory, to provide the accurate identification of biologicals—this condition is valid for biosimilars. Furthermore, as AEs can result from unintentional changes during manufacture, it is also suggested that the regulatory agencies remain informed of the batch number, to ensure proper traceability. However, most physicians only report the brand names, disregarding the batch number indication [18,52,58,63]. Furthermore, with the introduction of this new directive, the replacement of biosimilars (as they are marketed as branded products, i.e., never seen before) becomes more difficult, as it causes confusion and makes their acceptance difficult. Therefore, the type of name assigned to biosimilars will greatly affect their marketing, making the replacement of interchangeable biologicals (where permitted) less likely [52,54,62].

## 6. Implementation Model of Biosimilars in Hospital Settings

### 6.1. Economic Aspects of the Use of Biosimilars 

**Figure 5:**
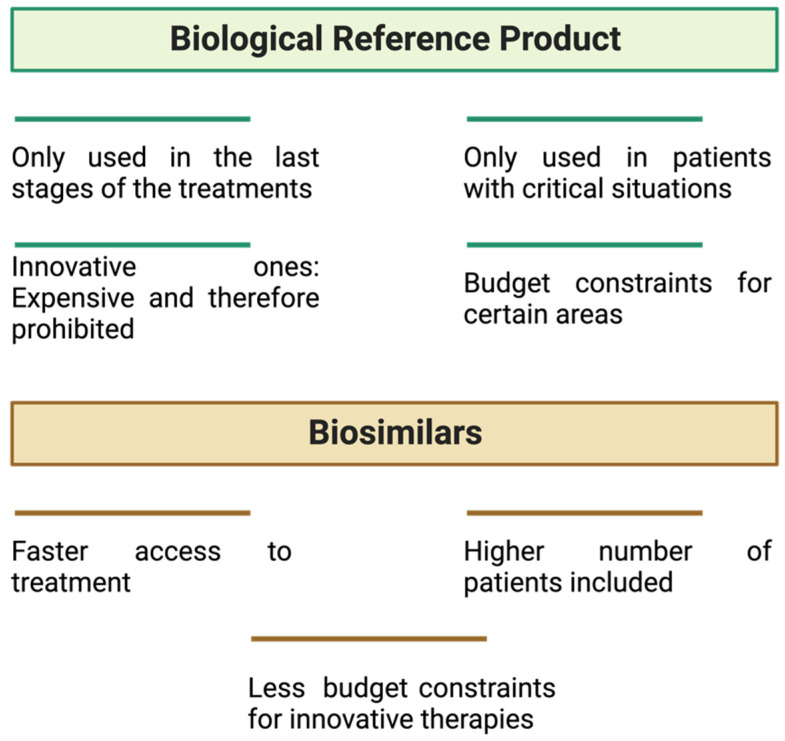
Main differences in terms of economic savings and public health impacts of using biological versus biosimilar medicines.

Considering the substantial increase in public expenditure on medicines (hospital and outpatient clinics) over the last few years (an increase of 30%), it is essential to avoid waste. Biosimilars are a valuable opportunity in this context, since they promote competition, generating greater accessibility, with an impact on sustainability and access, without altering the quality of care, which makes them more cost-effective therapeutic options [64]. The estimated cumulative savings in the EU and the US between 2016 and 2020 range from EUR 49 billion to EUR 98 billion. The introduction of biosimilars in the market can reduce healthcare costs and provide alternative treatment options. Additionally, their use can enhance patients’ ability to access biological therapies. Savings derived from the entry of biosimilars into the market can alleviate costly health budgets and open budget space for new treatment options. Furthermore, biosimilar intake may increase the patient’s access to biological therapies. The implementation of the use of biosimilars promotes a positive impact not only at an economic level but also at a social level, in terms of public health. In this context, the main differences in the use of biological and biosimilar medicines are outlined in Figure 5.

Europe has shown the strong adoption of biosimilar medicines, and, in recent years, these medicines have gained more than 7% of the organic market; this growth is related to the increase in the market of biosimilars in the areas of immunology and oncology [60]. With over 15 years of experience dealing with biosimilars in Europe and more than 2000 million patients/day of clinical experience, it can be said that biosimilars have a high standard of quality, safety, and efficacy, duly validated at the European level by the EMA [64].

It is known that the pharmacological class with the greatest burden for the National Health Service (NHS) is immunomodulators, with an approximate value of EUR 426 million (by 2020)—which corresponds to 31% of the total expenditure on medicines. Therefore, a reduction in the cost of these medicines allows for very significant savings for the NHS. 

### 6.2. What a Prescriber Needs to Know

Biosimilars are medicines subjected to high approval standards, so the probability of a problem occurring with any change in the manufacturing process is relatively low. However, healthcare professionals must be aware of the need for greater traceability of these products in the patient’s health records. It is not mandatory for them to know the particular modifications made during production. However, they should ensure that the best care is provided and that the medicines are used according to the required conditions, which includes using distinctive and product-specific names in health records and being aware of unexpected situations, such as adverse events [7]. For biosimilars to be prescribed, the medical community must fully understand them. Today, there are several resources to assist the patient in decision-making, such as up-to-date scientific evidence, regulatory requirements, available information on common inquiries, and discussions with specialists, among others [60]. 

Another important aspect that healthcare professionals should consider is the impact of the nocebo effect. This effect is related to patients having negative expectations regarding treatment, which consequently can lead to worse outcomes [61]. The nocebo effect is not related to the drug’s pharmacology, but to the fact that the patient associates the low cost of the product with a lack of efficacy [62]. Improved communication between health professionals and patients, avoiding the use of excessively technical language, can help to explain that the exchange of an RP for a biosimilar is safe and the biosimilar is equally effective [63]. For example, recently, the European Society of Medical Oncology issued statements to support healthcare professionals in the implementation of biosimilars in oncologic therapeutic management [59]. 

### 6.3. Patient Needs

Patients often express concerns about the efficacy and safety of biosimilars when compared to the reference biological products. Their low cost is one of the causes, since they fear that the health professionals’ decision is based only on this fact. To increase patient confidence, specific nomenclature and labeling transparency must be ensured [4]. 

Biosimilar availability for patients with arthritis/rheumatism has increased. These patients are, currently, one of the most representative groups using biological medicines. For this reason, the EULAR Standing Committee of People with Arthritis/Rheumatism in Europe (SCAPRE) published an article describing essential actions to help patients to understand biosimilars and therefore make informed decisions. One of the issues addressed in this document is related to the exchange, interchangeability, and replacement of reference biologicals and biosimilars, and it reports the need to establish easily understandable guidelines, created collaboratively with patients, that outline the expected actions and behaviors. Thus, patients will be able to make well-informed choices by evaluating the potential risks and benefits and discussing the advantages and drawbacks with their medical team [4]. 

## 7. Conclusions and Future Perspectives

It is widely agreed that the future will involve biotechnology—more specifically, biological medicines. Biosimilars are already widely recognized and established in clinical settings and are strong tools in helping people to gain greater access to treatments and medicines. As these medicines are much cheaper than the reference biologics, their use guarantees greater financial sustainability for the providing health systems. Being more cost-effective but identical in safety, quality, and efficacy, they will allow a greater number of patients to benefit from advanced therapies [49,57].

However, biosimilars present different challenges from generics, especially for manufacturers. The high costs of clinical development may be a drawback, as the process of manufacturing a biosimilar requires high investment and technical capacity. Another obstacle is the existing regulatory differences between the approval of biosimilars and the approval of generics (the regulatory framework for biosimilars is still very new in most markets). In addition, it is also necessary to gain the trust of health professionals and users, alleviating safety concerns, but, for this, it is necessary to invest heavily in marketing teams [17]. The market growth of these drugs requires constant regulatory and scientific updates, forcing companies to innovate (a scientific challenge) [1,17,19].

To maximize the earnings from the use of biosimilars, each stakeholder involved must carry out their obligations in the most effective manner possible. Physicians should better understand biosimilars to increase their confidence in prescribing them. Both physicians and patients should be aware of nocebo effects and implement strategies to overcome the limitations caused by negative expectations and possible decreases in treatment adherence [49,60]. Manufacturers must be able to quickly adjust to changes in the market and be competitively priced, ensuring product quality, supply sustainability, and the maintenance of PV systems. In other words, effective collaboration among all stakeholders involved is crucial in achieving biosimilar development. The primary aim is to provide patients with the clinical advantages of biological treatment while supporting the long-term viability of the healthcare system [17,49,60]. Therefore, the pharmaceutical industry is increasingly investing in new therapies, namely in the development of mAbs fragments, which have the same therapeutic targets as existing mAbs (the molecular weight is lower) [64]. 

According to regulatory agencies, the focus is on creating a solid and scientifically robust regulatory structure with the capacity to solve non-consensual points: the harmonization of nomenclature, the simplification of terminology, extrapolation, interchangeability, and automatic substitution. This will result in a smaller burden for biosimilar companies because the costs of their development are greater [1,2,18]. The latest EMA regulation on interchangeability has created harmonization in the EU, which is very important in promoting the adoption of biosimilars [61]. 

In this regard, despite the availability of guidelines for several years, there are still numerous concepts that require attention to provide an effective PV for biosimilar approval. It must be considered that post-approval safety monitoring programs do not have standardized requirements at present and vary among different manufacturers [5,12,24]. These programs are developed through conversations between the producer and regulatory agencies to determine which ones should be used. They must be covered by mechanisms capable of differentiating the AEs resulting from the biosimilar from those resulting from the RP.

The prospects and challenges for biosimilar companies are very much focused on future patent drops. However, at present, innovative biological drug companies, in addition to investing in new therapies and products, are also strategically focused on developing biobetters. These products, despite being considered innovative molecules, have very low development costs and associated risks, since data and studies on previous molecules already exist. As they are innovative molecules, they are entitled to patent and data exclusivity and do not have to wait for the patent on the original medicine to expire. Therefore, they can be marketed while maintaining a price equal to that of the RP, or a higher price may be established if they have superior quality [32,33,51].

The wide variety of challenges associated with biosimilars makes them currently an emerging and strategic field in the context of innovation and development in the pharmaceutical industry, but also a pillar in the sustainability of health systems, with an evident and relevant impact on the promotion of public health.

## Figures and Tables

**Table 1:** Main differences between biosimilars and generic medicines.

	Biosimilars	Generics	Ref.
**Product characteristics**	- Large complex molecules (up to 270,000 Da)	- Small and simple molecules (up to 300 Da)	[5]
**Production**	- Produced using live organisms (highly sensitive to manufacturing changes) - 5–9 years - High production costs	- Produced by chemical synthesis - 2–3 year - Lower production costs	[5,21]
**Structural comparison to reference medication**	- Highly similar to the RP: same amino acid sequence; - There may be differences in minor parts of the structure	- Structurally identical to the reference medicine	[4,5,12]
**Development**	- Comparability studies between the biosimilar and the RP	- Bioequivalence between the generic and the RP is evaluated	[4,22]
**Nomenclature**	- Rules vary from country to country	- Same chemical name (active ingredient) as the reference medicine	[12,16,18,23]
**Requirements for approval**	- Animal and clinical studies (toxicity, PK, PD, and immunogenicity)	- No animal or clinical studies (only bioequivalence studies) - The active ingredient must be identical in strength, dosage form, and route of administration	[3,5,12,23]
**Post-authorization activities**	- Pharmacovigilance (PV)	- Phase IV, risk management plan including PV	[9,18,24,25,26]
**Immunogenicity**	- Immunogenic	- Mostly nonimmunogenic	[23,24,25,27]
**Equivalence**	- Data must demonstrate, in each indication, that clinically significant differences, related to safety and efficacy, are not verified - Conclusive clinical studies may not be necessary for all indications	- Demonstration of bioequivalence is enough to grant all approved indications for the RP, without requiring any additional clinical studies	[5,12,18,23,26]
**Interchangeability and substitution**	- EMA see biosimilars to be scientifically interchangeable (2022), but any decision on the use of biosimilars is the mandate of the EU member states - FDA can designate a biosimilar interchangeable if a sponsor applies for this (but it is up to US State Law to permit substitution at pharmacies) - Automatic substitution is generally the decision of each country	- If permitted by state law, pharmacists may automatically substitute the generic for the reference medicine	[2,18,23,27,28]

**Table 2:** EMA’s regulatory guidelines related to the development and approval of biosimilars.

Topic	Title	Application
**Overarching**	- Guideline on similar biological medicinal products	**General—applies to all biosimilars**
**Quality**	- Guideline on similar biological medicinal products containing biotechnology-derived proteins as active substance: quality issues	
**Nonclinical and clinical**	- Guideline on similar biological medicinal products containing biotechnology-derived proteins as active substance: non-clinical and clinical issues	
**Annexes**	- Recombinant human erythropoietin - Recombinant GCSF - Recombinant human insulin - Recombinant human GH - INF-α and INF-β - Low-molecular-weight heparins - Monoclonal antibodies - Recombinant follicle-stimulating hormone	**Specific—product data requirements**

GCSF, granulocyte colony-stimulating factor; GH, growth hormone; INF-α, interferon alpha; INF-β, interferon beta.

## Data Availability

No new data were created.

## Abbreviations

AEs	Adverse Events
ADAs	Anti-Drug Antibodies
BD	Biodisponibility
CHMP	Committee for Medicinal Products for Human Use
CQAs	Critical Quality Attributes
EMA	European Medicine Administration
EEE	European Economic Area
EU	European Union
Fab	Fragment Antigen-Binding
Fc	Fragment Crystallizable
FcRn	Neonatal Fragment Crystallizable Receptor
FcγR	Fragment Crystallizable-Gamma Receptor
FDA	Food and Drug Administration
FIMEA	Finnish Medicines Agency
GCSF	Granulocyte Colony-Stimulating Factor
GH	Growth Hormone
ICH	International Conference of Harmonization
INFα	Interferon Alpha
INFβ	Interferon Beta
INN	International Naming System
RP	Reference Product
WHO	World Health Organization
mAb	Monoclonal Antibody
MA	Marketing Authorization
MS	Member State
PK	Pharmacokinetic
PD	Pharmacodynamics
PTMs	Post-Translational Modifications
PV	Pharmacovigilance
QbD	Quality-by-Design
QTPP	Quality Target Product Profile
SmPC	Summary of Product Characteristics
tRNA	Transfer RNA
